# Supplementary material for: Motor cortex can directly drive the globus pallidus neurons in a projection neuron type-dependent manner in the rat
Source: eLife. 2019 Nov 12;8:e49511. doi: 10.7554/eLife.49511 (PMC6863630; doi:10.7554/eLife.49511)
Supplement: Supplementary file 1. [file elife-49511-supp1.docx]

**Suppl. Table 1.** Primary antibodies used in this study

| Antigen | Host species | Supplier | Catalog number | RRID | Working dilution |
| --- | --- | --- | --- | --- | --- |
| Calbindin | mouse | Sigma | C9848 | AB_476894 | 1:4000 |
| Calbindin | rabbit | Frontier Institute | calbindin-Rb-Se-1 | AB_2571568 | 1:2000 |
| FoxP2 | rabbit | Abcam | ab16046 | AB_2107107 | 1:2000 |
| Lhx 6 | mouse | Santacruz | sc-271433 | AB_10649856 | 1:1000 |
| MOR | guinea pig | Millipore | AB1774 | AB_91022 | 1:5000 |
| MOR | rabbit | Neuromics | RA10104 | AB_2156525 | 1:1000 |
| PHA-L | goat | Vector | AS2224 | AB_2315141 | 1:2000 |
| Parvalbumin | mouse | Sigma | P3171 | AB_2313693 | 1:4000 |
| Parvalbumin | guinea pig | Synaptic Systems | 195004/16 | AB_2156476 | 1:5000 |

MOR, μ-opioid receptor; PHA-L, *Phaseolus vulgaris* leucoagglutinin

**Suppl. Table 2.** Secondary antibodies used in this study

| Secondary antibody | Host species | Supplier | Catalog no. | RRID | Dilution |
| --- | --- | --- | --- | --- | --- |
| anti-rat Alexa Fluor®488 | donkey | * | A21208 | AB_141709 | 1:500 |
| anti-mouse Alexa Fluor®546 | donkey | * | A10036 | AB_2534012 | 1:500 |
| anti-mouse Alexa Fluor®635 | goat | * | A31575 | AB_2536185 | 1:500 |
| anti-rabbit Alexa Fluor®405 | goat | * | A31556 | AB_221605 | 1:500 |
| anti-rabbit Alexa Fluor®635 | goat | * | A31577 | AB_2536187 | 1:500 |
| anti-guinea pig Alexa Fluor®405 | goat | Abcam | ab175678 | - | 1:500 |
| anti-guinea pig Alexa Fluor®594 | goat | * | A11076 | AB_141930 | 1:500 |
| anti-guinea pig Alexa Fluor®633 | goat | * | A21105 | AB_2535757 | 1:500 |

All antibodies are polyclonal. * Thermo Fisher Scientific (Waltham, MA), Abcam (Cambridge, UK)
