# Supplementary material for: Motor cortex can directly drive the globus pallidus neurons in a projection neuron type-dependent manner in the rat
Source: eLife. 2019 Nov 12;8:e49511. doi: 10.7554/eLife.49511 (PMC6863630; doi:10.7554/eLife.49511)
Supplement: Transparent reporting form [file elife-49511-transrepform.docx]

***eLife’s* transparent reporting form**

We encourage authors to provide detailed information *within their submission* to facilitate the interpretation and replication of experiments. Authors can upload supporting documentation to indicate the use of appropriate reporting guidelines for health-related research (see [EQUATOR Network](http://www.equator-network.org/%20)), life science research (see the [BioSharing Information Resource](https://biosharing.org/)), or the [ARRIVE guidelines](http://www.plosbiology.org/article/info:doi/10.1371/journal.pbio.1000412) for reporting work involving animal research. Where applicable, authors should refer to any relevant reporting standards documents in this form.

If you have any questions, please consult our Journal Policies and/or contact us: [editorial@elifesciences.org](mailto:editorial@elifesciences.org).

**Sample-size estimation**

- You should state whether an appropriate sample size was computed when the study was being designed
- You should state the statistical method of sample size computation and any required assumptions
- If no explicit power analysis was used, you should describe how you decided what sample (replicate) size (number) to use

Please outline where this information can be found within the submission (e.g., sections or figure legends), or explain why this information doesn’t apply to your submission:

To design the experiments, we considered similar kinds of earlier experiments from our own and other research groups. The number of animals used for each experiment is specified in the figure legends, as well as in the main text.

For immunohistochemical and neural tracing experiments (shown in Fig. 1, 2, 5, 6), we used 3 to 6 rats. First, we reviewed all samples and the sections, and confirmed all samples show qualitatively similar characteristics, then the samples from 2 to 4 animals were quantified.

For electrophysiological recording, we had to proceed two steps of experiments. First, we must confirm the unreported neural connections (Fig. 3). Second, we aimed to ask whether cell type dependence of the connection. Especially we must confirm whether or not the neuron group of low connection probability was observed just by chance, relatively larger number of neurons were recorded (Fig. 5).

**Replicates**

- You should report how often each experiment was performed
- You should include a definition of biological versus technical replication
- The data obtained should be provided and sufficient information should be provided to indicate the number of independent biological and/or technical replicates
- If you encountered any outliers, you should describe how these were handled
- Criteria for exclusion/inclusion of data should be clearly stated
- High-throughput sequence data should be uploaded before submission, with a private link for reviewers provided (these are available from both GEO and ArrayExpress)

Please outline where this information can be found within the submission (e.g., sections or figure legends), or explain why this information doesn’t apply to your submission:

As only one technical replicate, amplitude and shape of EPSC in individual neurons are measured for 10 repeats, and an averaged value (mean) is reported (Fig. 4, 5, 6).

For other results, all data are derived from biological replicates reported in the main text, namely, number of animals, neurons, brain sections, and region of interests.

For neuron tracer and viral vector injection experiments, the animals in which injection was not successfully located in an appropriate brain region were pre-excluded. These excluded samples are available for future studies aiming to other brain regions than reported in the manuscript

Putative cholinergic neurons in the globus pallidus were excluded from the analysis (Line 256-260).

**Statistical reporting**

- Statistical analysis methods should be described and justified
- Raw data should be presented in figures whenever informative to do so (typically when N per group is less than 10)
- For each experiment, you should identify the statistical tests used, exact values of N, definitions of center, methods of multiple test correction, and dispersion and precision measures (e.g., mean, median, SD, SEM, confidence intervals; and, for the major substantive results, a measure of effect size (e.g., Pearson's r, Cohen's d)
- Report exact p-values wherever possible alongside the summary statistics and 95% confidence intervals. These should be reported for all key questions and not only when the p-value is less than 0.05.

Please outline where this information can be found within the submission (e.g., sections or figure legends), or explain why this information doesn’t apply to your submission:

Raw data is presented in each figure. For comparison of two groups, Wilcox rank sum test was used.

For comparison of multiple groups (>2), one-way ANOVA and post-hoc Tukey test was applied.

For comparison of two distributions, Kolmogorov-Smirnov test was used.

For comparison of three distributions, Kruskal-Wallis test followed by multiple comparisons based on the Fisher Exact test with Bonferroni correction of p-values was used.

All p values were reported in each figure and Results.

All information is written in the Results and “Statistical Comparison” in Materials and Methods.

(For large datasets, or papers with a very large number of statistical tests, you may upload a single table file with tests, Ns, etc., with reference to sections in the manuscript.)

**Group allocation**

- Indicate how samples were allocated into experimental groups (in the case of clinical studies, please specify allocation to treatment method); if randomization was used, please also state if restricted randomization was applied
- Indicate if masking was used during group allocation, data collection and/or data analysis

Please outline where this information can be found within the submission (e.g., sections or figure legends), or explain why this information doesn’t apply to your submission:

This study does not contain any experiment with requirement for group allocation.

**Additional data files (“source data”)**

- We encourage you to upload relevant additional data files, such as numerical data that are represented as a graph in a figure, or as a summary table
- Where provided, these should be in the most useful format, and they can be uploaded as “Source data” files linked to a main figure or table
- Include model definition files including the full list of parameters used
- Include code used for data analysis (e.g., R, MatLab)
- Avoid stating that data files are “available upon request”

Please indicate the figures or tables for which source data files have been provided:

Source data files in a Microsoft Exel format are provided for Table 1, for Figures 1E, 2C, 3A2, 3B2, 3B3, 3B4, 4B, 4C, 4D, 4F, 5A, 5B, 5C, 5D, 6C, 6F, 8E, and also for Fig.1-figure supplement 3 and supplementary figure 1D.
